# Comment on “Comparison of clinical outcomes of open and closed cardioplegia sets used during cardiopulmonary bypass”

**DOI:** 10.1590/1806-9282.20251708

**Published:** 2026-05-08

**Authors:** Hüseyin Durmaz

**Affiliations:** 1 Konya City Hospital, Department of Cardiovascular Surgery & Konya, Türkiye.

Dear Editor,

I read with interest the article “Comparison of clinical outcomes of open and closed cardioplegia sets used during cardiopulmonary bypass,”^1^ recently published in the journal Revista da Associação Médica Brasileira by Dr. Padak et al. This study is an important and timely study in assessing the early clinical outcomes and inflammatory responses associated with the use of open and closed cardioplegia sets.

The data presented in the study clearly demonstrate the impact of preferred cardioplegic sets on patient morbidity. In this regard, the recommendations offered to improve the predictability of clinical outcomes and inflammatory responses are quite important. However, I believe there are several points to be addressed in this study.

The effects of steroids on the suppression of the inflammatory response to cardiopulmonary bypass and on postoperative outcomes have been evaluated in multicenter studies^2,3^. There is an interaction between intraoperative dexamethasone treatment and the risk of inflammation and postoperative C-reactive protein^4^. Information regarding steroids is not available in the current study. Steroid treatment should be mentioned because it can alter postoperative C-reactive protein levels.

Hyperglycemia is a side effect of steroids^5^. The failure to mention steroid treatment in the current study reduces the reliability of the postoperative glucose results. Postoperative drainage is important because it can affect postoperative outcomes. One criticism of the study is the lack of postoperative drainage information about the patients.

In conclusion, Dr. Padak et al.’s study offers a valuable starting point by addressing the important issues with cardioplegic sets. We eagerly await further research in this area and hope the authors will consider these recommendations in their future studies.

Thank you for the opportunity to provide feedback on this important study.

## Data Availability

The datasets generated and/or analyzed during the current study are available from the corresponding author upon reasonable request.
